# Plasma Cell Balanitis Unresponsive to Topical Corticosteroids: Combined Efficacy of Tacrolimus and Emerging Evidence for Underrecognized Intrinsic Resistance to Corticosteroids

**DOI:** 10.7759/cureus.81306

**Published:** 2025-03-27

**Authors:** Moeno Miyajima, Noritaka Oyama, So Inamura, Yoshiaki Imamura, Minoru Hasegawa

**Affiliations:** 1 Dermatology, University of Fukui, Fukui, JPN; 2 Urology, University of Fukui, Fukui, JPN; 3 Surgical Pathology, University of Fukui, Fukui, JPN

**Keywords:** corticosteroid resistance, plasma cell balanitis, topical corticosteroids (tcs), topical tacrolimus, zoon's balanitis

## Abstract

Plasma cell balanitis (PCB), also known as Zoon’s balanitis, is a chronic, benign inflammatory dermatosis affecting the glans penis, mainly occurring in uncircumcised middle-aged men. Currently, there has been no recommended and standardized treatment. Topical corticosteroids are the most common first-line therapy in clinical practice but often account for the primary inefficacy relapse and adverse events, particularly in cases requiring long-term use due to the high transcutaneous absorption in the penile skin. A series of recent case reports may suggest topical calcineurin inhibitors as a promising alternative, offering significant improvement in corticosteroid-resistant cases. In this paper, we describe a 61-year-old uncircumcised male patient with a one-year history of asymptomatic erosive erythema on the penis, which was initially unresponsive to topical corticosteroids. Lesional skin biopsy histologically confirmed a diagnosis of plasma cell balanitis. Combined treatment of a preceding topical corticosteroid with 0.1% tacrolimus ointment led to a dramatic clinical response within two weeks of treatment, followed by almost complete resolution after six weeks. This approach enabled the maintenance of the treatment efficacy with a weak-potency corticosteroid throughout the clinical course. Recurrence occurred with reduced application frequency, but the daily use of the same regimen rapidly restored favorable disease control. We discuss the potential difficulty in managing PCB, with particular interest in the relatively high frequency of corticosteroid resistance in nearly half of the cases reported to date. This paper also updates the possible mechanisms of action and clinical benefits of topical calcineurin inhibitors in plasma cell biology.

## Introduction

Plasma cell balanitis (PCB), also known as Zoon's balanitis, first described in 1952 [1], is a chronic, benign, inflammatory dermatosis localized on the glans penis and prepuce. It predominantly affects middle-aged and older men, with a higher prevalence in uncircumcised men. Analogous conditions include plasma cell vulvitis in females and plasma cell cheilitis in oral mucosa, collectively termed plasmacytosis circumorificialis (PLC), that occur around periorifices of the body [2-4]. Provoking factors include poor hygiene (e.g., irritation with urine or colonization of gram-negative bacteria), trauma, friction, and excessive moisture [5]. Surgical approaches, including circumcision, may completely resolve in some cases, but often they argue about overtreatment due to their invasive nature [6,7].

Topical therapies are the mainstay, but no standardized protocols have currently been available for PCB. Topical corticosteroids are most frequently employed, albeit with a relatively high relapse rate [8]. Moreover, prolonged use of potent corticosteroids may trigger an increasing risk of adverse events, particularly on the thin and sensitive penile skin, due to the high transcutaneous absorption. Recent case series have explored the potential benefit of topical calcineurin inhibitors (CIs), tacrolimus and pimecrolimus, in cases with corticosteroid-resistant PCB [9]. The favorable efficacy of CIs raises a question regarding the underlying mechanisms contributing to inadequate or poor corticosteroid responses in the disease.

We herein present such a case of long-standing, recalcitrant PCB that was unresponsive to topical corticosteroid monotherapy but showed dramatic improvement with the additive use of topical tacrolimus. This combined treatment approach allowed for maintaining the treatment efficacy with a weak potency corticosteroid throughout the clinical course. Our case report provides an updated perspective on the intrinsic difficulty in managing PCB in clinical practice, with a comprehensive literature review.

## Case presentation

**Figure 1:**
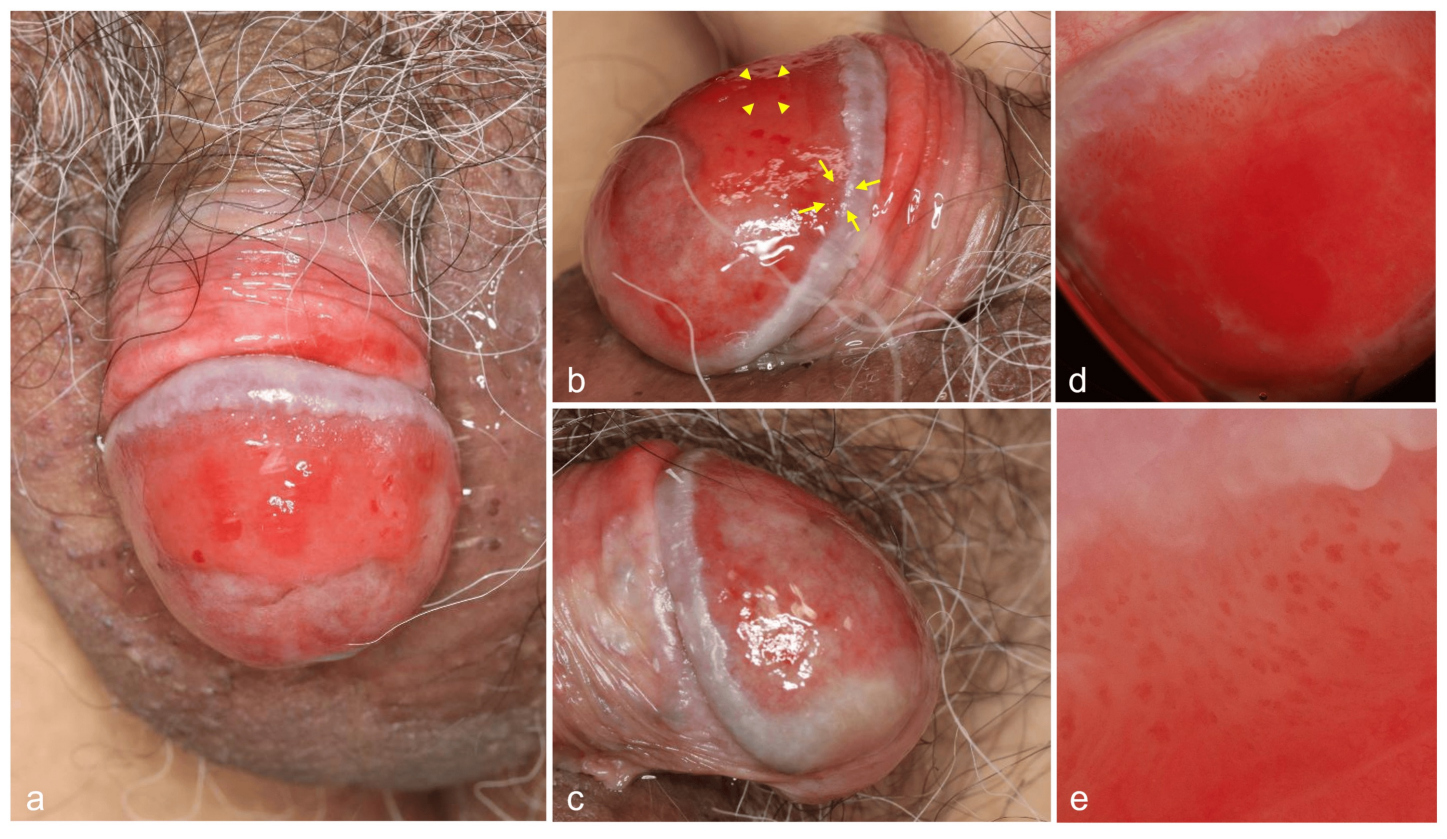
Clinical findings Clinical pictures showed a shiny red, irregular erythema fully covered with erosions in the dorsal aspect of the glans penis and penis (a, b, c). Dermoscopic findings illustrated homogenous, uniform reddish areas exhibiting a glomerular pattern (d), surrounded by dilated irregular vessels forming a serpentine pattern (e), as assessed by a DZ-D100 dermoscope (Casio Computer Co., Ltd., Tokyo, Japan; x10 magnification). Arrowheads and arrows indicate the sites of the former glomerular pattern and the latter serpentine pattern in dermoscopic findings, respectively.

A 61-year-old, uncircumcised Japanese male patient presented with a one-year history of erythema on the glans penis. General physicians gave topical corticosteroids that were almost unhelpful, with a gradual extension of the skin lesions. On examination, a shiny red, irregular erythema fully covered with erosions was observed in the dorsal aspect of the glans penis (Figures 1a-1c). Although initially asymptomatic, he subsequently experienced a mild burning sensation in the affected skin. His inguinal lymph nodes were unpalpable. He was otherwise healthy, without sexual intercourse or a particular drug history. Dermoscopic examination revealed homogenous, uniform reddish areas surrounded by dilated, irregular vessels. The former reddish areas showed a glomerular pattern, characteristic of homogeneous capillary dilation, while the dilated irregular vessels exhibited a serpentine pattern (Figures 1d, 1e).

**Figure 2:**
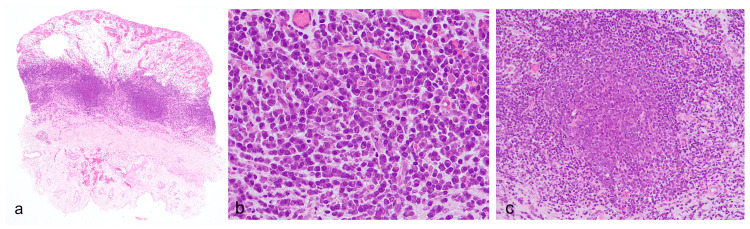
Histological findings Skin biopsy showed superficial ulceration with intense interstitial edema and hypervascularization in the upper dermis (a, loupe magnification, H&E). The mid dermis exhibited a dense infiltration of monotonous plasma cells without atypia, focally reminiscent of lymphoid follicles (b, x400, H&E; c, x200, H&E).

Routine laboratory investigations, including infectious screening, such as syphilis, chlamydia, gonorrhea, and *Escherichia coli* (*E. coli*), yielded no significance. Skin biopsy showed an erosive surface lacking epidermis with intense interstitial edema and hypervascularization in the upper dermis (Figure 2a).

In the mid dermis, dense and homogeneous infiltration of non-atypical plasma cells was present, focally resembling lymphoid follicles (Figures 2b, c). Immunostaining showed no biased expression of immunoglobulin lambda and kappa light chains, with almost negligible levels of IgG4-expressing cells, highlighting the polyclonality of the plasma cell population (data not shown). The overall statement suggests the diagnosis of plasma cell balanitis.

**Figure 3:**
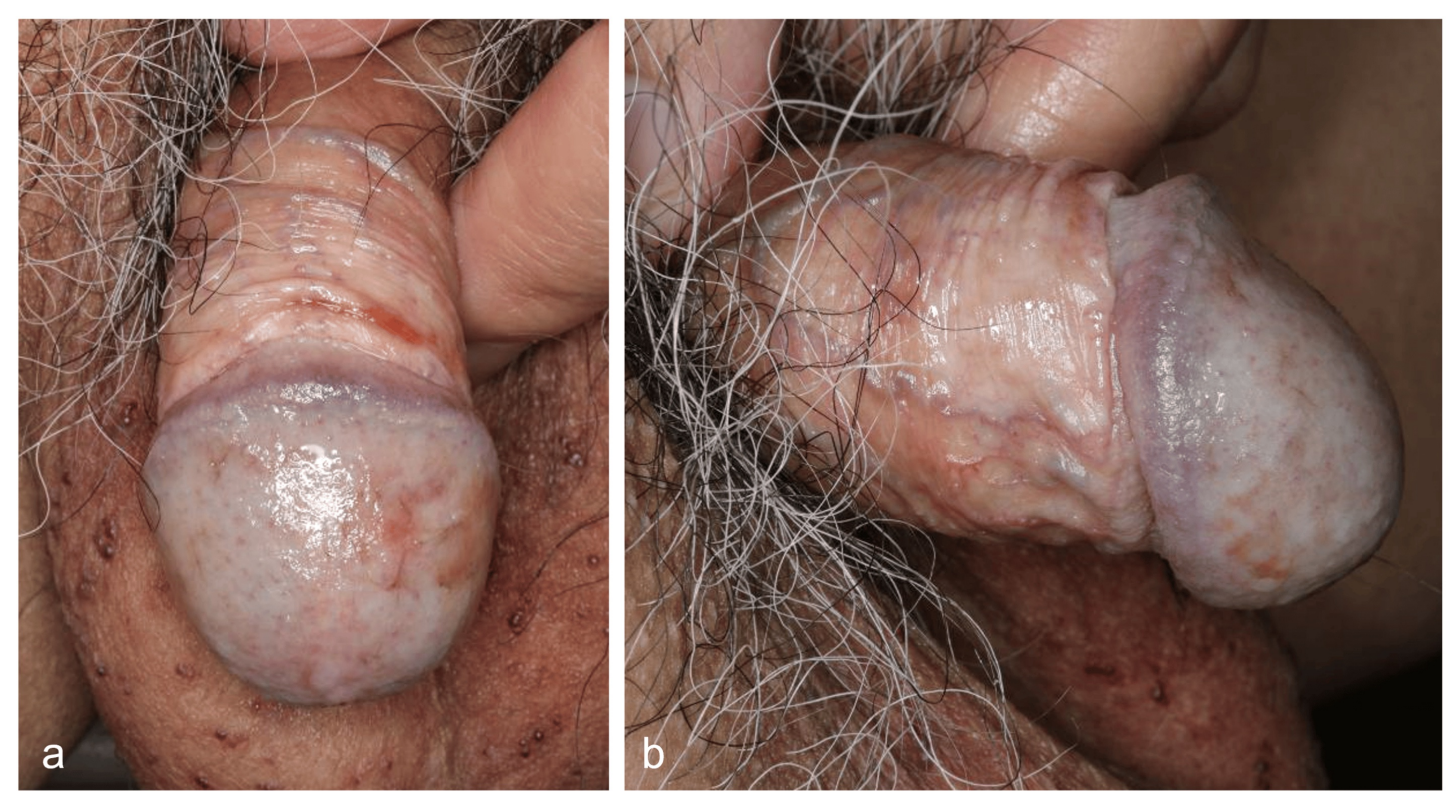
Clinical findings after combined topical treatment After six weeks of the topical combined treatment, erosion was epithelialized without symptoms (a, b). No recurrence was observed over six months of follow-up.

During the diagnostic process, he continued a provisional treatment with 0.05% clobetasone butyrate ointment alone, which had no clinical improvement, consistent with the previous treatment response. Subsequently, a combined use of 0.1% topical tacrolimus ointment overlay and the corticosteroid twice daily was started. Dramatic improvement was obtained at two weeks of treatment, characterized by re-epithelialization and disappearance of erythema, as well as fewer local symptoms. After six weeks of treatment, he achieved almost complete resolution of the erosive skin without symptoms. No adverse effects were noted throughout the treatment course. Clinical follow-up over six months confirmed maintaining a favorable disease remission (Figures 3a, 3b).

## Discussion

Plasma cell balanitis often displays a debilitating clinical course that is primarily unresponsive to topical corticosteroids or relapses, particularly upon steroid tapering. Uncontrollable disease may carry a potential risk of local malignancy, although only three reported cases with malignant transformation in preexisting PCB have been documented thus far [10,11]. Prolonged, constant use of potent corticosteroids on genital skin and mucosa may cause an increasing risk of adverse reactions, such as skin thinning, irritation, and telangiectasia, which may complicate differentiation from active PCB lesions or infection. In addition, topical corticosteroids may be attributed, in part, to the downregulation of local glucocorticoid receptors, resulting in drug resistance, a phenomenon reported in other genital dermatoses [12,13]. Thus, the limitation of corticosteroid monotherapy raises the potential necessity for alternative strategies in managing refractory PCB, like in our case.

Topical CIs, including tacrolimus and pimecrolimus, have emerged as steroid-sparing agents in a variety of chronic inflammatory dermatoses, including lichen planus, psoriasis, and atopic dermatitis [14]. Tacrolimus exerts its therapeutic effect by mainly inhibiting T cell activation and suppressing overproduction of pro-inflammatory cytokines, including IL-2, TNF-α, and IFN-γ [15,16]. These pro-inflammatory cytokines play synergistic and complementary roles in the expression of IL-6, a pivotal cytokine in the overall plasma cell biology, including its proliferation, survival, and differentiation [15], while T cells can also produce IL-6 in a subset-dependent manner [17]. These cytokine cascades promoting IL-6 production may contribute to the expansion and maintenance of plasma cell populations, characteristic of PCB pathology [18]. Notably, topical tacrolimus offers distinct advantages in genital skin and mucosa due to its superior transcutaneous penetration (molecular weight=822) and efficacy without causing skin atrophy and telangiectasia, which are commonly associated with prolonged corticosteroid use.

Our case demonstrated a significant improvement in long-standing, corticosteroid-resistant PCB with the combined use of a weak-potent corticosteroid and tacrolimus. Considering the potential for irritation with topical tacrolimus, particularly on eroded skin, we initially implemented a combined therapy with corticosteroids to effectively minimize local irritation. This approach enabled us to maintain the treatment's efficacy even with a weak-potency corticosteroid throughout the clinical course, without escalating the potency. This favorable result with topical tacrolimus is in agreement with previous reports demonstrating the rescue efficacy of CIs in PCB who failed to respond to any of the primary treatments [8,19].

Despite the favorable treatment response in our case, the long-term safety and efficacy of topical CIs in PBC require further reliable validation. In addition, combination therapy with topical corticosteroids should be carefully considered based on disease presentation or severity and prior treatment history. Other research interests would include estimating whether the primary use of topical CIs provides superior clinical benefit compared to that of corticosteroids alone, as well as determining the optimal dosage, appropriate treatment duration, and potential predictors for selecting CIs to refine evidence-based management for the disease.

## Conclusions

Our case demonstrates the therapeutic advantage of topical tacrolimus ointment for the management of corticosteroid-resistant PCB. The anti-inflammatory and non-anthropogenic properties of CIs, combined with their suitability for sensitive genital mucocutaneous areas, may position them as a promising therapeutic option for PCB, particularly in cases with poor responses to the primary treatment, including topical corticosteroids. Further studies warrant well-designed, controlled trials not only to validate the efficacy of topical CIs in corticosteroid-resistant cases but also to apply their expected efficacy as a primary treatment modality.
